# Blockade of fructose transporter protein GLUT5 inhibits proliferation of colon cancer cells: proof of concept for a new class of anti-tumor therapeutics

**DOI:** 10.1007/s43440-021-00281-9

**Published:** 2021-05-29

**Authors:** Jakub Włodarczyk, Marcin Włodarczyk, Marta Zielińska, Bartłomiej Jędrzejczak, Łukasz Dziki, Jakub Fichna

**Affiliations:** 1 Department of Biochemistry, Medical University of Lodz, Mazowiecka 6/8, 92-215 Lodz, Poland; 2 Department of General and Colorectal Surgery, Medical University of Lodz, Żeromskiego 113, 90-549 Lodz, Poland

**Keywords:** Colorectal cancer, Fructose, GLUT5, *N*-[4-(methylsulfonyl)-2-nitrophenyl]-1,3-benzodioxol-5-amine

## Abstract

**Background:**

Despite the fact that colorectal cancer (CRC) is one of the most commonly diagnosed cancers in men and women, its current treatment remains unsatisfactory and therefore novel studies proposing new approaches are necessary. A high sugar diet is believed to promote carcinogenesis. Fructose is absorbed from the gastrointestinal tract by members of the glucose transporter family—GLUT. The aim of the study was to characterize the expression of GLUT5 at mRNA level in CRC patients. Moreover, our goal was to elucidate the molecular role of GLUT5 in CRC and assess whether GLUT5 inhibitor may affect the viability of colon cancer cells.

**Methods:**

The expression of GLUT5 at mRNA level was characterized based on 30 samples from resected colorectal cancers and 30 healthy colonic mucosa specimens from surgical margins. The inhibitory effect of *N*-[4-(methylsulfonyl)-2-nitrophenyl]-1,3-benzodioxol-5-amine (MSBNA) was assessed on a colon cancer cell line, HT-29, and normal colon epithelium cells—CCD 841 CoN Cells.

**Results:**

GLUT5 expression was found in 96.7% of cancer specimens and only in 53.3% of healthy mucosa fragments. In cancer tissue, real-time PCR analysis showed almost 2, fivefold (*p*< 0.001) increase of GLUT5 mRNA expression level compared with the healthy intestinal mucosa. GLUT5 inhibitor, MSNBA (10 µM) significantly decreased the viability of colon cancer cells, while barely affected the viability of normal colon epithelium cells.

**Conclusions:**

Our study suggests that a strong focus should be put on GLUT5 and its inhibitors for both diagnostic and therapeutic purposes in CRC.

## Introduction

Colorectal cancer (CRC) is the third most commonly diagnosed cancer in men (746,000 new cases per year) and the second in women (614,000 new cases per year). It is also the second leading cause of cancer-related deaths in Western countries [1]. Many people with CRC do not experience any symptoms in the early disease stages or these symptoms are nonspecific (weakness, fatigue, diarrhea/constipation), therefore it is diagnosed mainly in the advanced stage [2]. Late diagnosis in CRC leads to an increase in mortality and morbidity rates and although new detection and treatment strategies are regularly proposed, CRC still poses a great threat to human health [3].

Although currently, the most effective strategies in CRC treatment are surgery, radiotherapy, and chemotherapy, their outcomes still remain unsatisfactory [4]. Moreover, despite recent advances in the management of CRC, metastatic disease remains challenging and patients are rarely cured. The 5-year survival rate for patients with CRC is 64%, whereas in metastatic CRC it is only 14% [5]. Recently, the role of targeted therapy in the management of patients with CRC has gained more attention. Multiple studies proposed new treatment approaches such as inhibitors of angiogenesis, EGFR‐ or BRAF mutation–targeted therapies, and other strategies, including immunotherapy [6].

Apart from age, male sex, and hereditary factors, diet is the most important risk factor which is responsible for 35% of CRC incidence [7]. Consumption of sugars is increasing worldwide [8] and its association with cancer is still unknown, yet possible. It has been suggested that a high sugar diet may promote carcinogenesis by stimulation of insulin and insulin-like growth factor-I synthesis [9], induction of oxidative stress [10] or promotion of weight gain [11].

About 10% of calories contained in the Western diet are supplied by fructose (approximately 55 g/day) [12] which is absorbed from the gastrointestinal tract through passive transport across cell membranes by members of the glucose transporter family—GLUT. The GLUT family consists of 14 members divided into three major classes based on sequence homology and substrate selectivity [13]. GLUT5 (class II) and GLUT2 (class I) are the major fructose transporters in the body. The former is the sole transporter specific for fructose with no ability to transport glucose or galactose.

Recently, new design options for novel therapeutics against obesity, diabetes, and cancer have been opened due to the synthesis of a specific inhibitor of human GLUT5 [14]. For example, in a human breast cancer cell line MCF7, MSNBA decreased competitively inhibited GLUT5 fructose uptake and, in consequence, cell viability.

The aim of the study was to determine the expression of GLUT5 at mRNA level in CRC patients in different stages of cancer. Moreover, our goal was to elucidate the molecular role of GLUT5 in CRC and assess whether GLUT5 inhibitor may affect the viability of colon cancer cells.

## Materials and methods

### Patients

This prospective clinical study was performed in adult CRC patients, hospitalized at the Department of General and Colorectal Surgery at the Medical University of Lodz, Poland. We enrolled 30 patients in different stages of CRC. All approvals required to perform this study were obtained from the Committee of Bioethics of Medical University of Lodz (RNN/831/14/KB). The study enrolled only the patients hospitalized with the diagnosis of CRC who gave their written and informed consent to participate. The case report form for each patient qualified for the study including demographic data, progression, and stage of CRC (Table 1).

**Table 1:** Patients’ demography and cancer characteristics

Age	60.9 years
Sex	
Male	*N* = 15
Female	*N* = 15
Localization	
Sigmoid	*N* = 12
Descending colon	*N* = 6
Transverse colon	*N* = 6
Ascending colon	*N* = 6
Grade	
G1	*N* = 7
G2	*N* = 16
G3	*N* = 7
Stage	
II	*N* = 10
III	*N* = 10
IV	*N* = 10

### Collection of colonic biopsies and quantification of GLUT5 expression

To quantify GLUT5 expression, forceps tissue samples from resected colorectal cancers and healthy colonic mucosa from surgical margins were collected immediately after surgical operation. After isolation of tissue, the biopsy specimens were immediately frozen and kept at −80 °C until processing.

### RNA isolation

Total RNA isolation was performed using commercially available TRIsureTM (Bioline, Australia). Colon samples were minced and homogenized in TRIsureTM. After centrifugation and phases separation, the aqueous phase was mixed 3:1 (v/v) with isopropanol and loaded on the column. The subsequent steps were conducted according to the manufacturer’s protocol. The quality and quantity of RNA were estimated spectrophotometrically with BioPhotometer Plus (Eppendorf, Germany). The RNA was characterized with A260/A280 ratio, which was in the range of 1.70–2.00.

### Reverse transcription and quantitative real-time PCR

cDNA synthesis was performed with a High-Capacity cDNA Reverse Transcription Kit (Applied Biosystems, USA) in accordance with the manufacturer’s protocol. Total RNA (1 μg) was used in reverse transcription reaction with the following incubation steps: 25 °C for 10 min, 37 °C for 120 min and 85 °C for 5 min for RNA. The quantification of mRNA was performed using the real-time PCR method with FAM dye-labeled TaqMan^®^ probes (Applied Biosystems, USA). The reaction mixture consisted of cDNA, TaqMan^®^ Master Mix II, no UNG, TaqMan^®^ Assays and RNase-free water in a total volume of 10 μl. The cycle parameters for TaqMan® Assays were as follows: initial denaturation at 95 °C for 10 min, followed by 40 cycles of sequential incubations at 95 °C for 15 s and at 60 °C for 1 min. The obtained results were normalized to the expression of GAPDH (glyceraldehyde 3-phosphate dehydrogenase) for the studied genes. All experiments were performed as triplicates. The real-time PCR was performed using Master Realplex4s (Eppendorf, Germany). The fluorescent dye emission was a function of the cycle number. The initial amount of the temple was evaluated as a Ct parameter. The Ct value corresponded to the threshold cycle number at which PCR amplification reached a significant threshold. The relative expression level was calculated as 2 ^− ∆Ct^ × 1000. The results are expressed as the number of examined mRNA copies per 1000 copies of mRNA for GAPDH.

### Cell culture

The colon cancer cell line HT-29 and normal colon epithelium CCD 841 CoN cells were obtained from the American Type Culture Collection (ATCC HTB-38 and CRL-1790). The cells were cultured in Dulbecco’s modified Eagle’s medium supplemented with 10% FBS 1% l-glutamine and 1% Penicillium, Neomycin, and Streptomycin (Gibco, Life Technologies). The cells grew at 37 °C in a humidified atmosphere and 5% CO_2_, according to the standards procedures. For all experiments, colon cell lines were seeded onto 96-well plates and cultured for 2 or 3 days. Prior to the following analysis, the expression of GLUT5 receptor on both HT-29 and CoN cells on a transcript level was confirmed.

### Cell viability

The cell viability was analyzed using cell MTT Cell Viability Assay Kit. The test was performed according to the manufacturer's protocol. The cells were seeded in 96-well tissue culture plates to reach optimal density (48–72 h). Then the cells were incubated with (5 mM fructose;10 mM fructose—considering the maximum possible plasma concentration at postprandial condition; 2 mM MSNBA—GLUT5 inhibitor; control group) and 10 µl of MTT for 4 h at 37 °C. Next, 200 µL of DMSO was added directly into the medium in each well and pipetted up and down several times to dissolve the formazan salt. The final volume in the well was 300 µl. The absorbance signal was measured on a spectrophotometer at 570 nm, while background absorbance was measured at 630 nm. The whole assay was performed in six technical replicates and in three biological replicates.

### Statistical analysis

The statistical analysis was performed in Prism 8.0 (GraphPad Software Inc). *p* values < 0.05 were considered statistically significant. A Shapiro–Wilk test was used to test the determined normality of the distribution of variables; continuous variables were expressed as mean ± SD (standard deviation). The statistical significance between the groups was determined using unpaired *t* test or two-way ANOVA followed by Tukey’s post hoc test. The Spearman’s rho correlation coefficient was used to determine the correlation between the GLU5 mRNA expression level and the cancer grade and stage, which was determined according to the American Joint Committee on Cancer (AJCC) criteria.

## Results

### mRNA levels

**Fig. 1:**
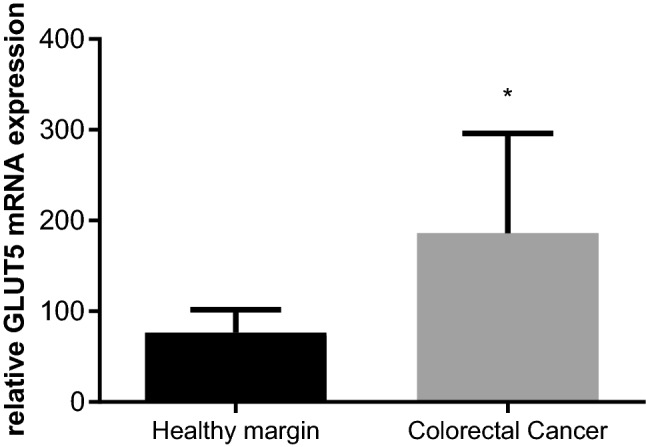
GLUT5 expression at the mRNA level in samples from CRC tissue and from healthy margin colon mucosa. Data are expressed as mean ± SD. *Significant difference at *p* < 0.001 relative to the healthy margin. Statistical significance was determined using unpaired *t* test

We analyzed the expression of GLUT5 at the mRNA level in 30 CRC samples and 30 healthy intestinal mucosa biopsy specimens from surgical margins. Reference GAPDH gene expression was detectable in all 60 analyzed samples. Noteworthy, GLUT5 expression was found in 96.7% of cancer specimens and only in 53.3% of healthy mucosa fragments. In the cancer tissue, the real-time PCR analysis showed almost a 2.5-fold increase of GLUT5 mRNA expression level compared with healthy intestinal mucosa (Fig. 1). The mean relative expression of GLUT5 in CRC was significantly higher (186.1 ± 110.1), whereas its expression in healthy margins was lower (76.38 ± 25.5; *P* < 0.001; *t* = 3.911).

Thirty cases of colorectal cancer were analyzed using Spearman's correlation analysis to determine the relationship between GLUT5 mRNA expression level and cancer grade and stage. As shown in Table 2, GLUT5 expression level showed a significant positive correlation with cancer grade.

**Table 2:** Correlation between GLUT5 relative expression in cancer tissue and tumor grade and stage assessed by Spearman’s rho correlation

GLUT5 relative gene expression	Grade			Stage		
	G1 (*n* = 7)	G2 (*n* = 16)	G3 (*n* = 7)	II (*n* = 10)	III (*n* = 10)	IV (*n* = 10)
Mean ± SD	130.84 ± 65.05	168.25 ± 103.19	277.59 ± 128.86	133.16 ± 62.05	189.89 ± 132.67	232.06 ± 119.00
*p*	0.0309			0.0663		
Correlation rho	0.3946			0.3396		

### Cell viability

A preliminary study confirmed the expression of the GLUT5 receptor on both HT-29 and CoN cells on a transcript level. The relative expression of GLUT5 on HT-29 and CoN cells was, respectively, 0.31 and 0.23.

**Fig. 2:**
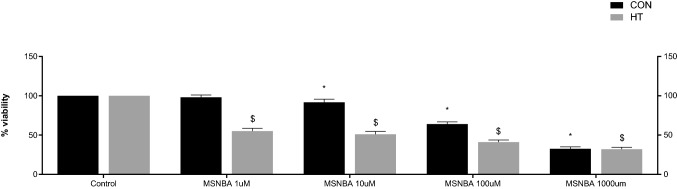
Cytotoxicity evaluation of MSNBA in HT-29 and CoN cells. Cells were exposed to MSNBA at various concentrations for 24 h, and then cell viability was detected by MTT assay. Data are expressed as mean ± SD (*n* = 6). Statistical significance from the two-way ANOVA, Tukey’s post hoc test: ^*^p < 0.001 versus CoN control group; ^$^*p* < 0.001 versus HT-29 control group

A 24 h incubation of HT-29 cells with GLUT5 inhibitor, MSNBA, significantly decreased their viability (51% decrease with 10 µM and 55% with 1 µM MSNBA), while CoN cells were only slightly affected by these concentrations of MSNBA (respectively 92 and 98%), as assessed by two-way ANOVA (effect of MSNBA: *F*_4,50_ = 1278.8, *p* < 0.001; effect of HT-29 cells: *F*_1,50_ = 1125.94, *p* < 0.001; interaction between MSNBA and HT-29: *F*_4,50_ = 206.09, *p *< 0.001), followed by Tukey’s post hoc test (Fig. 2). IC50 value for HT-29 cells was significantly lower than for CoN cells (0.25 µM ± 0.08 vs. 12.51 µM ± 1.41; *p* < 0.001; *t* = −15.08).

**Fig. 3:**
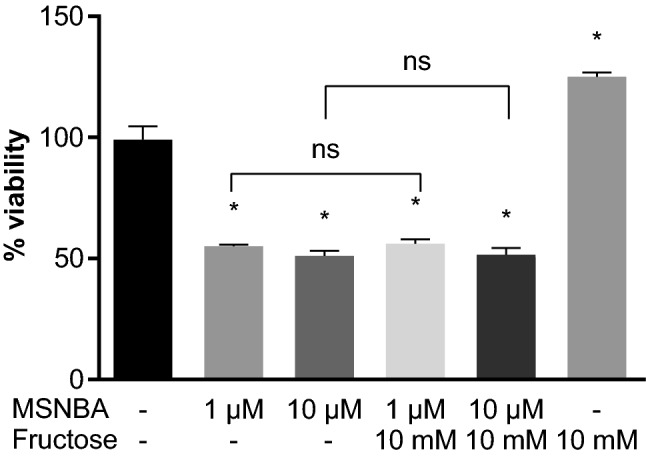
Cytotoxicity evaluation of MSNBA and fructose in HT-29. Cells were exposed to MSNBA and fructose at various concentrations for 24 h, and then cell viability was detected by MTT assay. Data are expressed as mean ± SD (n = 4). Statistical significance from the two-way ANOVA, Tukey’s post hoc test: *p < 0.001 versus control group

The addition of 10 mM fructose with MSNBA in the same concentrations did not affect HT-29 cell viability (respectively, 52 and 54%), as assessed by two-way ANOVA (effect of MSNBA: *F*_2,18_ = 254.164, *p* < 0.001; effect of fructose: *F*_1,18_ = 7.403, *p* = 0.014; interaction between MSNBA and fructose: *F*_2,18_ = 10.519, *p* < 0.001), followed by Tukey’s post hoc test. However, when the cells were incubated with 10 mM fructose alone, their viability increased to 125% (Fig. 3).

**Fig. 4:**
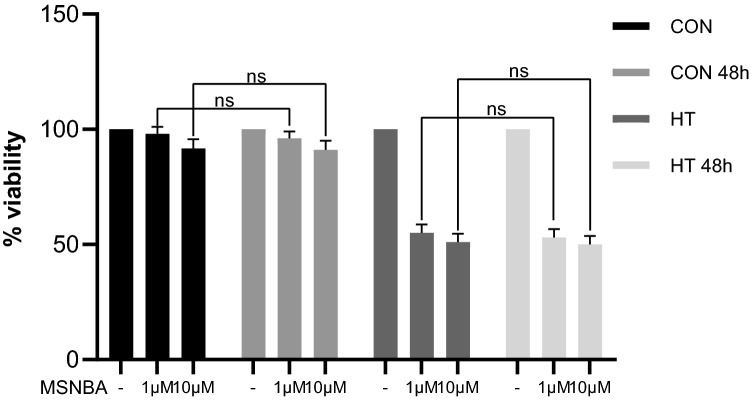
Cytotoxicity evaluation of MSNBA and time of incubation in HT-29. Cells were exposed to MSNBA at various concentrations for 24 or 48 h, and then cell viability was detected by MTT assay. Data are expressed as mean ± SD (*n* = 6). Statistical significance from the two-way ANOVA followed by Tukey’s post hoc test

The incubation for 48 h with MSNBA did not change the viability of HT-29 cells (50% viability—10 µM MSNBA; 53% viability—1 µM MSNBA, compared with 24 h incubation) (Fig. 4). CoN cells displayed similar viability to 24 h incubation—91% after 10 µM MSNBA and 96% viability after 1 µM MSNBA exposure for 48 h. As assessed by two-way ANOVA (effect of time: *F*_1,60_ = 2.5, *p* = 0.1187; interaction between time and HT-29: *F*_1,60_ = 0.043, *p* = 0.8358; interaction between time and MSNBA: *F*_2,60_ = 0.9, *p* = 0.3972; interaction between time, HT-29 and MSNBA: *F*_2,60_ = 0.7, *p* = 0.4825), followed by Tukey’s post hoc test.

## Discussion

The aim of the study was to characterize the expression of GLUT5 in healthy colon mucosa and colon cancer tissue, as well as to assess whether GLUT5 inhibitor, *N*-[4-(methylsulfonyl)-2-nitrophenyl]-1,3-benzodioxol-5-amine (MSNBA), might decrease the viability of colon cancer cells in vitro. We found that the GLUT5 expression was higher in carcinomatous colon tissues than in the healthy resected margins. Our study also showed that MSNBA has a significant effect on reducing colon cancer cell viability while barely affecting healthy intestinal epithelial cells.

The GLUT family consists of 14 members, which are extensively described in the literature. Many of them play a significant physiological role in the gastrointestinal tract [15, 16], being responsible for absorption of monosaccharides in the intestine, which is crucial for caloric intake. The activity of the GLUT family is adjusted in accordance with food supply, food composition, and energy demand in diverse physiological and pathophysiological situations. In addition, GLUT5 was proved to be present in a normal colon epithelium [17].

The literature confirms that GLUT5 overexpression may be linked to pathologies, such as hypertension, renal diseases, and hepatic dysregulations [18]. It was also found that GLUT5 is expressed in numerous cancerous tissues, particularly breast, renal, liver, and testicular cancer [17]. Breast cancer cells have been shown to express GLUT5 at higher mRNA and protein levels than healthy breast tissue. A similar pattern of GLUT5 expression was observed in renal cell carcinoma compared to healthy cells [19]. Moreover, in in vitro studies Caco2 cells and highly proliferative cancer cells were characterized by GLUT5 expression, which may be seen as as a potential marker of malignancy or high proliferation rate [20]. Our study indicated that GLUT5 is expressed in colon carcinoma and its expression is significantly increased in comparison to healthy colonic tissue. Moreover, the GLUT5 expression is positively correlated with cancer grade. This is the first study to evidence the possible role of GLUT5 in colon carcinogenesis and indicates the transporter’s diagnostic and therapeutic potential.

It needs to be noted that the role of fructose in cancer cell biology and the importance of GLUT5 in fructose-related cellular processes have been already indicated [21, 22]. For example, studies showed that, due to increased metabolism, cancer cells increased calories/energy demand and the usage of fructose and other monosaccharides escalated in these cells [23]. Moreover, recent reports have suggested that overexpression of GLUT5 in cancer tissues is positively correlated with fructose uptake [24]. This may be related either to the presence of GLUT5 or to an increased usage of fructose: the former leads to greater use of fructose by neoplastic cells, while the latter results in a higher abundance of GLUT5. In most of the tumor cells overexpressing GLUT5, the rate of fructose uptake is exacerbated, indicating that fructose may be the preferred substrate providing the energy required for growth and proliferation. More recently, GLUT5 has been linked not only to cancer growth and energy expenditure but also to cancer cell migration induced by metabolic changes and the development of drug resistance [25]. This effect is mediated by AKT1 and AKT3 activation and miR-125b-5p downregulation. What is more, GLUT5 silencing with small interfering RNA attenuated mesenchymal marker expression and migratory activity in drug-resistant colon cancer cells. Additionally, the treatment with 2,5-anhydro-D-mannitol, a competitive inhibitor of fructose uptake, resensitized chemoresistant cancer cells to oxaliplatin and 5-fluorouracil. It was thus proposed that GLUT5 expression after chemotherapy can serve as a new marker to indicate metabolic change-induced migration and drug resistance development. This also strongly suggests that the inhibitors of GLUT5 may constitute a future therapeutic approach in colorectal or other cancers.

Excessive consumption of fructose, for which GLUT5 is the sole transporter, is associated with an increased risk of CRC [26]. A recent study was focused on the effects of daily oral administration of high-fructose corn syrup (HFCS) in adenomatous polyposis coli (APC) mutant mice, which are predisposed to develop intestinal tumors [27]. According to Goncalves et al., the HFCS-treated mice showed a substantial increase in tumor size and tumor grade. Within the tumors, fructose was converted to fructose-1-phosphate, leading to activation of glycolysis and increased synthesis of fatty acids that support tumor growth. Blocking the fructose transporter (GLUT5) may inhibit this process and therefore stop colon cancer growth. In the second part of our study, we evidenced that inhibition of GLUT5 by MSNBA efficiently decreased the viability of colon cancer cells, which may be used to design an experimental treatment of CRC as well as to prevent CRC growth. However, the future investigation of MSNBA and its impact on CRC is still necessary.

Lately, a novel modulator of GLUT5 has been proposed. As a ligand-activated transcription factor, liver X receptor α (LXRα) might provide novel pharmacologic strategies for the selective modulation of GLUT5 activity in the treatment of both metabolic disease and cancer [28]. It was proved to have a strong impact on the expression of GLUT5 in the intestine. Additionally, a whole-cell-based GLUT5 assay system amenable to high-throughput ligand screening has recently become available, enabling an accelerated discovery of future GLUT5 inhibitors [29]. While we have proved that GLUT5 inhibitor, MSNBA, decreases colon cancer cells viability, novel GLUT5 modulators may have a stronger impact on carcinogenesis in the intestines.

## Conclusions

Fructose, accounting for ~ 5–15% of daily calorie intake, is linked with various morbidities, including tumorigenesis, obesity, diabetes, as well as heart and kidney diseases [30]. Studies revealed a fructose correlation with increased risk of breast cancer progression and metastasis [31]. In pancreatic tissue, it has been confirmed that fructose can be utilized for the synthesis of nucleic acid and to promote cell proliferation by cancer cells [32]. In the future, the mentioned morbidities may constitute a global health problem, as they are correlated with the increasing worldwide fructose intake. Therefore, novel therapeutics are required. Our study suggests that a strong focus could be put on GLUT5 and its inhibitors for both, diagnostic and therapeutic purposes in CRC. What follows, this subject matter warrants further investigations.
